# Alcohol and the Medical Profession

**Published:** 1914-11

**Authors:** 


### Alcohol and the Medical Profession.

The development of the mass majority of the people against the reign of King Alcohol has been steady, and a long line of constant gain for absolute temperance, and nowhere has this been more marked and more rapid than in the medical profession, and among the medical journals, since the ice was broken, and the start upward was made.

No law is stronger than public opinion behind it; there is a referendum that operates whether we formally desire it or not.

Practically, the medical journals are a unit in the battle against the ravages of intemperance until, within the past decade, the majority of the medical writers and publications alike have come to teach that alcohol is not indispensable as a stimulant; science is teaching that there are other agents which will do the work as well, if not better, and that, even were the former claims as to the necessity of alcohol true, still the fact remains that the other ravages of strong drink constitute too heavy a price to pay for the benefits.

The argument that innocent victims of sickness would suffer from the withdrawal of the drug from the pharmacopeia may be met by the counter condition that the greatest suffering from intemperance falls most heavily upon the innocent, and that the sum of such suffering far outweighs that which would result from the dropping of alcoholic stimulation from the practice of medicine; so that, even were the most extravagant claims as to the good from the prescription of this potent drug facts, still the benefits would not begin to counterbalance its evils.

Alcohol is being driven from its last stronghold, that of the plea of medical necessity, and every doctor should prepare to take his place on the firing line for a better humanity.

G. H. B.

---

Dr. J. S. Lankford, of San Antonio, Texas, was one of the distinguished speakers at the American Life Convention, which recently held its annual meeting in Dallas. The subject of the doctor's paper was, "The Heart in Life Insurance."

